# Large‐Scale Production of Secretome From Human Dental Pulp Stem Cells for Articular Cartilage Regeneration

**DOI:** 10.1155/sci/4200051

**Published:** 2026-07-18

**Authors:** Lucía Bravo-Baranda, Lara Milián, María Sancho-Tello, Mauro Llop-Miguel, Irene Monleón-Guinot, Cristina Martínez-Ramos, José Manuel Morales-Tatay, María Fe Mínguez-Rey, Manuel Mata

**Affiliations:** ^1^ Department of Pathology, University of Valencia, Valencia, Spain, uv.es; ^2^ INCLIVA Biomedical Research Institute, Valencia, Spain, incliva.es; ^3^ Department of Surgery, University of Valencia, Valencia, Spain, uv.es; ^4^ Orthopedic Surgery and Traumatology Service, University Clinical Hospital, Valencia, Spain; ^5^ Biomedical Research Networking Center on Bioengineering, Biomaterials and Nanomedicine (CIBER-BBN), Zaragoza, Spain, ciber-bbn.es

**Keywords:** cartilage regeneration, human dental pulp stem cells (hDPSCs), mesenchymal stem cells (MSCs), osteoarthritis, secretome

## Abstract

**Introduction:**

Articular cartilage degeneration can lead to a progressive loss of joint function and, eventually, total joint failure. The use of mesenchymal stem cells (MSCs) for cartilage regeneration has proven effective; however, the difficulty of their clinical application has prompted research into other strategies, such as using MSC secretome instead of cells themselves. Our aim was to generate a culture system to obtain large amounts of MSC secretome with a controlled and known composition and the potential to induce articular cartilage regeneration.

**Methods:**

Human dental pulp stem cells (hDPSCs) embedded in alginate/agarose beads were cultured in a bioreactor with constant agitation and periodic culture medium exchange. The secretome was collected and characterized according to known chondral markers (TGF‐β1, SERPINE1, miR‐140, miR‐675, miR‐23a, miR‐204, miR‐211, and miR‐337‐5p). Metabolomic and proteomic analyses were carried out for detailed characterization of the collected secretome. The chondrogenic potential of the collected secretome was tested in an in vitro model developed using human primary chondrocytes cultured in 3D on alginate‐agarose scaffolds.

**Results:**

Our system allowed us to obtain 1200 mL of secretome from intensive cultures of hDPSCs. The cells cultured on the platform remained viable and in an active anabolic state, secreting large amounts of prochondrogenic mediators. Variability between batches of secretome was low, and the collected secretome induced primary chondrocyte differentiation in vitro.

**Conclusions:**

Genomic, metabolomic, and proteomic data indicate that hDPSCs on the platform proliferate and acquire a chondrocyte‐like phenotype. These cells secrete mediators that define a microenvironment favorable for chondral regeneration. This is further supported by evidence found in the in vitro differentiation model. Our platform allows the production of large volumes of secretome with controlled composition and chondrogenic induction potential. This is a necessary preclinical study for the subsequent analysis of the secretome obtained using in vivo experimental models.

## 1. Introduction

Diarthroses are complex and essential joints in the human body, allowing for a wide range of bone movements while absorbing impact and facilitating load bearing. Joint architecture is highly organized and includes several key elements, such as articular cartilage, a special type of hyaline cartilage characterized by the lack of perichondrium, which limits the tissue’s regenerative capacity [1].

Articular cartilage degeneration is an irreversible process initiated by either inflammatory processes, such as osteoarthritis (OA), or by trauma. In most cases, it leads to a progressive loss of joint function and, eventually, complete joint failure, which significantly affects patients’ quality of life [2, 3]. Therefore, diseases related to articular cartilage constitute a major social and healthcare challenge, mainly affecting developed countries with an aging population [4].

In recent years, significant efforts have been made to develop therapeutic strategies that mitigate or slow down the degeneration process of articular cartilage. These approaches include microdrilling, autologous chondral transplantation, as well as the use of various biochemical factors and biomaterials, among others [4]. However, these therapies are not sufficiently effective in many cases, and some patients require prosthesis implantation. Although joint prostheses significantly improve mobility and alleviate pain in patients whose symptoms severely impacted their quality of life, they also present notable limitations related to their short lifespan, which limits their use, especially in young patients [5, 6].

The usefulness of mesenchymal stem cells (MSCs) has been demonstrated in vivo studies and in clinical trials for OA, both by direct injection into the joint cavity and by implantation in injured cartilage. They have shown to have a beneficial effect in the patients studied, with a reduction in pain and inflammation [7]. Among the different origins of the MSCs used, we highlight human dental pulp stem cells (hDPSCs), which are ectomesenchymal cells easily isolated from dental pulp tissue and have demonstrated chondrogenic potential both in vitro and in vivo [8, 9].

One limitation of the clinical use of MSCs lies in their ability to generate tumors or transmit infectious diseases. However, the pro‐regenerative effect of these cells appears to be due to the secretion of a set of anti‐inflammatory and pro‐regenerative biomolecules, known as the secretome, that modify the tissue microenvironment and generate a favorable environment for regeneration. Therefore, the use of the secretome instead of the MSCs themselves is becoming increasingly important [10, 11]. However, the results obtained using MSCs’ secretome are heterogeneous, which may be due to various factors, such as the culture system used, which often consists of 2D systems that do not allow for the control of cell dynamics. Another important aspect to consider is the influence of the tissue microenvironment on MSCs’ behavior since a prochondrogenic environment is essential for these cells to release the factors necessary for chondral regeneration [12]. Finally, a better characterization of the secretome is needed to identify the factors involved in the chondral regeneration process and thus standardize a product suitable for clinical use [13].

In this work, we aimed to develop a culture system that would allow us not only to produce a large amount of secretome of known composition in a reproducible and scalable manner but also to characterize its composition at the protein and metabolomic levels. To achieve this, we cultured hDPSCs embedded in alginate/agarose hydrogel beads at high cell density for 4 weeks in a chondrogenic culture medium to stimulate their chondrogenic differentiation. We designed a bioreactor for continuous replacement of the culture medium and collected hDPSCs’ secretome during the 4 weeks of culture for metabolomic and proteomic characterization. Finally, the chondrogenic potential of the collected secretome was analyzed in an in vitro experimental model.

## 2. Materials and Methods

### 2.1. Experimental Design

**Figure 1:**
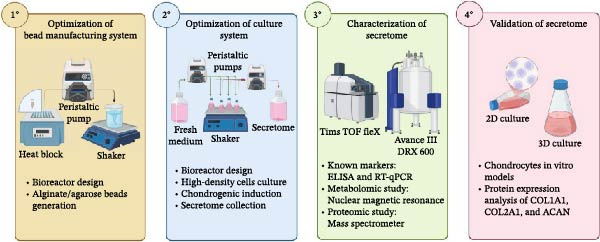
Summary of the experimental design. The main processes performed in each experimental phase are indicated: (1°) Optimization of bead manufacturing system; (2°) Optimization of culture system; (3°) Characterization of secretome; and (4°) Validation of secretome. Created in BioRender. Bravo‐Baranda, L. (2025).

Figure 1 summarizes the experimental design followed in this study. We organized the work into four successive phases, with the aim of developing a culture system that allows us to generate large volumes of MSCs secretome with chondrogenic potential and a known composition.

In the first phase, we designed a bioreactor for the rapid and reproducible production of alginate/agarose beads with embedded hDPSCs. This study included five different patients. We analyzed the generated beads at the morphometric and biomechanical levels while optimizing the hydrogel composition, which allowed us to generate highly homogeneous beads.

In the second phase, we designed and manufactured another bioreactor for the culture of hydrogel beads containing high cell density and with constant culture medium turnover. To optimize culture conditions, we analyzed the distribution and viability of hDPSCs embedded and cultured in the beads. Then, we induced chondrogenesis in the bead‐embedded hDPSCs by using a specific culture medium. We collected the culture medium for 4 weeks and studied the expression of chondrogenesis‐related markers, such as transforming growth factor beta 1 (TGF‐*β*1) and SERPINE1, as well as the presence of miRNAs associated with the acquisition of the chondral phenotype, such as miR‐23a, miR‐204, miR‐211, miR‐337‐5p, miR‐140, and miR‐675.

In the third phase, we characterized the secretome composition. For this purpose, aliquots of the secretome were collected during the 4 weeks of culture and pooled into the “early secretome” (weeks 1 and 2 of culture) and “late secretome” (weeks 3 and 4 of culture). The secretome was then studied to analyze known chondrogenesis‐related proteins using high‐resolution mass spectrometry (HRMS) and chondrogenesis‐related metabolites using proton nuclear magnetic resonance (^1^H‐NMR).

In the fourth and final phase, the chondrogenic induction capacity of the collected secretome was studied in vitro using primary cultures of human chondrocytes, isolated from articular cartilage, cultured in both 2D monolayers and 3D alginate/agarose hydrogel beads with embedded cells. This is a model well established by us and other research groups for the study of chondrogenesis in vitro [8, 14–16].

The chondrocytes were incubated with the secretome for 4 weeks, and the expression of COL1A1, COL2A1, and ACAN was studied by immunofluorescence (IF), whereas cell morphology was assessed by fluorescent F‐actin staining. The chondrogenic potential of the collected secretome was compared with that of the chondrogenic medium.

### 2.2. Cell Culture and Differentiation

hDPSCs and chondrocytes were obtained from human donors. This study was conducted in accordance with the Declaration of Helsinki and applicable local regulatory requirements and laws and was approved by the Ethics Committee of the University Clinical Hospital of Valencia (Spain; identification code 2016/27, approved in April 2019) and by the Ethics Committee of the University of Valencia (Spain; identification code H1548322921081, approved in February 2019), respectively. Five different donors were included in this study. All donors signed informed consent.

hDPSCs were isolated from donor third molars, as previously described [9]. After tooth extraction, the dental pulp was gently removed under sterile conditions using cow horn forceps with a small excavator and transferred to a tube containing Hank’s solution (Gibco, Madrid, Spain). Samples were then cut into small fragments with a scalpel and digested with dispase for 2 h at 37°C in a 5% CO_2_ and 95% atmosphere. The supernatant was removed, and 0.1% type‐IV collagenase (Sigma‐Aldrich, Merck KGaA, Darmstadt, Germany) was added for 15 min, followed by centrifugation at 400×*g* for 10 min. The supernatant was removed, and cells were seeded in 25 cm^2^ flasks and expanded with α‐Minimum Essential Medium (MEM) proliferation medium supplemented with 10% fetal bovine serum (FBS), 1 mM L‐glutamine (all 3 from Gibco, Thermo Fisher Scientific, Waltham, MA, USA), 1% Penicillin‐Streptomycin (Pen/Strep), and 1% amphotericin B (both from Euroclone S.p.A., Pero, Milan, Italy).

Human chondrocytes were isolated from the articular cartilage of healthy donors by successive digestion with hyaluronidase, pronase, and collagenase, as previously reported [14]. Cells were expanded in Dulbecco’s Modified Eagle Medium (DMEM) proliferation medium supplemented with 10% heat‐inactivated FBS, 1% MEM non‐essential amino acids (NEAA) (all 3 from Gibco, Thermo Fisher Scientific, Waltham, MA, USA), 50 µg/mL L‐ascorbic acid, 1% sodium pyruvate (both from Sigma‐Aldrich, Merck KGaA, Darmstadt, Germany), 1% Pen/Strep, and 1% amphotericin B.

Chondral differentiation was induced by incubating cells during 4 weeks with either hDPSCs secretome, diluted 1:4 in high‐glucose DMEM containing 1% Pen/Strep, 1% amphotericin B, or with chondrogenic differentiation medium consisting of a serum‐free high‐glucose DMEM differentiation medium containing 1% insulin‐transferrin‐selenium (ITS) (both from Gibco, Thermo FisherScientific, Waltham, MA, USA), 5 μM aminocaproic acid, 10 ng/mL TGF‐β1 (both from Sigma‐Aldrich, Merck KGaA, Darmstadt, Germany), 10^−7^ M dexamethasone, 0.2% hyaluronic acid (HA) (both from Thermo Scientific Chemicals, Waltham, MA, USA), 50 µg/mL L‐ascorbic acid, 1% Pen/Strep, 1% amphotericin B, and 1% MEM NEAA, as previously described [17].

### 2.3. Hydrogel Generation and Cell‐Embedded Beads

A 6% sodium alginate stock solution was prepared as previously described [18]. Briefly, sodium alginate (alginic acid sodium salt from brown algae; Sigma‐Aldrich, Merck KGaA, Darmstadt, Germany) was dissolved with continuous stirring in a sterile solution containing 40 mM HEPES (Gibco, Thermo Fisher Scientific, Waltham, MA, USA) and 300 mM NaCl and prewarmed to 65°C. After cooling the alginate solution to room temperature (RT), the pH was adjusted to 7.4. The solution was subsequently autoclaved and stored at 4°C until use. In addition, an 8% ultra‐low melting point type IX agarose stock solution was prepared by dissolving the appropriate amount of agarose in sterile calcium‐free phosphate‐buffered saline (PBS; Sigma–Aldrich, Merck KGaA, Darmstadt, Germany), as previously described [19]. The agarose stock solution was warmed at 37°C on a hot plate until completely dissolved, and the alginate stock solution was also prewarmed to 37°C.

**Figure 2:**
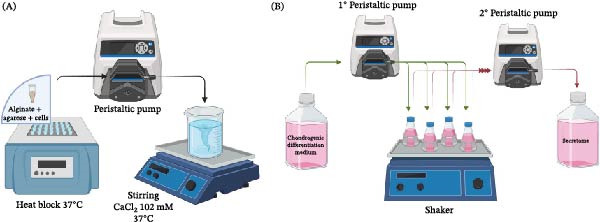
Generation of hydrogel beads with embedded cells, and secretome collection. Panel (A) shows schematic representation of the hydrogel bead generation process for 3D embedding of hDPSCs. An alginate–agarose suspension containing isolated cells was maintained at 37°C using a heat block before being extruded using a peristaltic pump into a 102 mM CaCl_2_ solution at 37°C with constant stirring, which allowed the formation of ionic cross‐linked hydrogel beads that embedded the cells. Panel (B) shows schematic representation of the 3D dynamic continuous medium exchange system used for secretome collection. Cultures were maintained under intermittent shaking and continuously perfused with fresh medium using a peristaltic pump. The secretome was collected from the outflow tract for further analysis. Created in BioRender. Bravo‐Baranda, L. (2025).

To generate cell‐embedded hydrogel beads, cultured hDPSCs or chondrocytes were trypsinized, and the isolated cells were resuspended in the alginate solution (2 × 10^6^ cells/mL hydrogel) and subsequently mixed with the agarose stock solution at 37°C at different ratios. For alginate crosslinking, a 102 mM CaCl_2_ solution in sterile water was used, prewarmed to 37°C, and maintained under continuous stirring. Beads with embedded isolated cells were then formed by dripping the hydrogel mixture onto the CaCl_2_ solution using a Multiflow peristaltic pump (Art.No. 4901, LAMBDA Laboratory Instruments, Baar, Switzerland), which allowed precise control of the bead size (Figure 2, panel A). A silicone tube measuring 55 cm long, 1 mm inner diameter, and 3 mm outer diameter, coupled with a 10‐μL pipette tip, was used to generate the polymer droplets. The beads were incubated at RT for 30 min, followed by an additional 30 min at 4°C, to induce agarose gelation and stabilize the hydrogel.

Once the cell‐embedded hydrogel beads were obtained, the beads were transferred to a flask, and culture medium was added at a ratio of 50 mL of medium per mL of hydrogel used to generate the beads. The flask was incubated in a humidified atmosphere with 5% CO_2_ at 37°C in a bioreactor specifically designed for this purpose (Figure 1, panel B), consisting of two multichannel peristaltic pumps (G100‐2J/DG‐4‐B, Longer Precision Pump Co., Ltd., Baoding, Hebei, China) that constantly supplied and removed the culture medium to four flasks, each containing 300 beads. Each pump had four channels, and we used the first pump to add the culture medium and the second one to remove it, so the speed of these devices had to be synchronized. Initially, the pumps were placed inside the cell incubator, but we detected a significant temperature increase due to their operation, reaching 39°C, so we decided to remove the pumps from the incubator and keep them outside thereafter. We connected the first pump, which introduced the medium into the flasks, to a tube long enough to warm the culture medium. The culture medium exchange rate was adjusted to 10% of the volume of each flask daily.

### 2.4. Mechanical Compression Tests

The mechanical properties of the hydrogels were evaluated by uniaxial compression testing using a Zwick Z0.5 TN testing machine (ZwickRoell GmbH & Co. KG, Ulm, Germany) equipped with a 5 N load cell. The samples were compressed to a 15% strain at a deformation rate of 30%/min, with an initial preload of 3 mN. All measurements were carried out at RT, with the samples immersed in a drop of PBS to maintain hydration.

The elastic modulus was calculated from the slope of the linear region of the stress–strain curve using testXpert software (ZwickRoell GmbH & Co. KG, Ulm, Germany). Each hydrogel sample was measured only once as mechanical loading caused irreversible water loss, which would significantly alter subsequent measurements.

### 2.5. Cell Viability Assessment

Cell viability was evaluated using the Live/Dead Cell Viability Kit for 3D and 2D Cell Cultures (Merck KGaA, Darmstadt, Germany) following the manufacturer’s instructions. Briefly, cell‐embedded beads were incubated with the staining solution for 1 h at 37°C, followed by washing with PBS. Fluorescence images were acquired using a Leica DM2500 fluorescence microscope (Leica Microsystems, Wetzlar, Germany). Quantification was performed with QuPath software using automated image analysis [20]. The percentage of dead cells was calculated as the ratio of propidium iodide‐positive cells (dead or dying cells) to the total number of nuclei (stained with Hoechst 33342).

### 2.6. Staining of Cell‐Embedded Beads With HematoxylinEosin and Periodic Acid‐Schiff (PAS)

Cell‐embedded beads were collected and fixed with 4% paraformaldehyde at 4°C for 4 h, followed by an overnight wash at RT in a solution containing 100 mM sodium cacodylate and 50 mM BaCl_2_ (pH 7.4). After standard dehydration steps, the beads were embedded in a 1:1 mixture of paraffin wax (Sigma‐Aldrich, Merck KGaA, Darmstadt, Germany) and absolute ethanol and maintained overnight at 37°C. Then, samples were immersed for 1 h in pure paraffin wax, and paraffin blocks were formed at RT, in a dry environment using silica gel as a desiccant (Sigma–Aldrich, Merck KGaA, Darmstadt, Germany). Sections of 5 µm thickness were obtained, deparaffinized, rehydrated, and stained with hematoxylin–eosin or with PAS, following routine procedures. After staining, sections were dehydrated and mounted using Entellan (Sigma–Aldrich, Merck KGaA, Darmstadt, Germany). Image acquisition was performed using a Leica DM4000 microscope equipped with a DFC digital camera (Leica, Madrid, Spain).

### 2.7. Secretome Collection

After embedding the hDPSCs in hydrogel beads, they were cultured for 1 week in α‐MEM proliferation medium (described above) to allow cell expansion. After that initial proliferation phase, the beads were transferred to a chondrogenic differentiation medium and maintained for 4 weeks. They were cultured at 37°C in a humidified atmosphere with 5% CO_2_ and 95% air, with intermittent shaking at 100 rpm for 30 min every 2 h. Based on previous publications, we generated a continuous medium exchange system (Figure 2, panel B) where the daily collected volume of exchanged medium represented 10% of the total volume of the culture flask, as mentioned above [21]. The daily collected conditioned medium was first centrifuged at 3000×*g* for 20 min to remove cell and hydrogel debris, and the supernatant was frozen at −20°C. After 2 or 4 weeks of culture, these frozen conditioned media were thawed and pooled into two groups (up to 2 weeks of culture for the early secretome, weeks 3 and 4 for the late secretome), and then 100‐fold concentrated by centrifugation using Amicon Ultra centrifugal filters (MWCO 30 kDa; Millipore, Merck KGaA, Darmstadt, Germany) at 4000×*g* for 20 min. Finally, the secretome was collected from the upper fraction of the pooled supernatant (>30 kDa) and was frozen at −80°C until use.

For metabolomics studies, an aliquot of the collected conditioned culture medium was not concentrated with Amicon filters and therefore also contained metabolites <30 kDa.

### 2.8. Analysis of miRNA Expression of Known Markers

miRNAs with a known relationship with chondrogenesis were analyzed by real‐time quantitative polymerase chain reaction (RT‐qPCR) from the secretome released by cells. Total RNA was extracted using the TRIzol LS Reagent (Thermo Fisher Scientific, Waltham, MA, USA), following the manufacturer’s instructions. For reverse transcription of miRNAs, the TaqMan MicroRNA Reverse Transcription Kit (Cat. No. 4366596, Thermo Fisher Scientific, Waltham, MA, USA) was used according to the protocol provided by the manufacturer.

Quantitative PCR was performed using the TaqMan Fast Advanced Master Mix (Cat. No. 4444558, Thermo Fisher Scientific, Waltham, MA, USA) and the TaqMan MicroRNA Assays specific for hsa‐miR‐23a (000399), hsa‐miR‐204 (000508), hsa‐miR‐211 (000514), hsa‐miR‐337‐5p (002156), has‐miR‐140 (002234), and has‐miR‐675 (002005). All reactions were carried out in duplicate on a 7900HT Real‐Time Thermocycler (Applied Biosystems, Madrid, Spain).

The synthetic spike‐in control cel‐miR‐39 (Assay ID: 10620310, Thermo Fisher Scientific, Waltham, MA, USA) was added prior to RNA extraction and used for normalization. Relative expression levels were calculated using the comparative ΔΔCt method.

### 2.9. Quantification of TGF‐β1

Levels of human TGF‐β1 were quantified in the secretome using the DuoSet ELISA kit (Cat. No. DY240‐05), in combination with the DuoSet ELISA Ancillary Reagent Kit 1 (Cat. No. DY007B) (both from R&D Systems, Bio‐Techne, Minneapolis, MN, USA) was used according to the manufacturer’s instructions. Briefly, 96‐well microplates were coated with the capture antibody diluted in PBS and incubated overnight at RT. After washing and blocking, 100 μL of samples or standards was added to each well and incubated for 2 h at RT. The plates were then incubated sequentially with the detection antibody and with Streptavidin‐HRP for 120 and 20 min, respectively, with washes between incubations. The signal was developed using the substrate solution, and the reaction was stopped after 20 min with the stop solution. Absorbance was measured at 450 nm with wavelength correction at 540 nm using a VICTOR3 Multilabel Plate Reader (PerkinElmer, Waltham, MA, USA). A standard curve from 2000 to 31.3 pg/mL was generated with recombinant human TGF‐β1, and the sample concentrations were interpolated accordingly.

### 2.10. Quantification of PAI‐1/SERPINE1

Human plasminogen activator inhibitor‐1 (PAI‐1, also called SERPINE1) levels were quantified using the Human PAI‐1 ELISA Kit (Cat. Nos. BMS2033 and BMS2033TEN, Thermo Fisher Scientific, Waltham, MA, USA), following the manufacturer’s instructions. Samples were prediluted 1:50 in assay buffer (10 μL sample + 490 μL buffer). Then, 50 μL of the diluted sample or standards and 50 μL of assay buffer were added to the wells, followed by 50 μL of biotin conjugate. After 2 h of incubation at RT on a microplate shaker, wells were washed and incubated for 1 h with Streptavidin‐HRP. After additional washes, 100 μL of TMB substrate solution was added to each well and incubated for 10 min in the dark. The reaction was stopped by adding stop solution, and absorbance was measured at 450 nm with correction at 620 nm using a VICTOR3 Multilabel Plate Reader (PerkinElmer, Waltham, MA, USA). A standard curve from 5000 to 78 pg/mL was used to interpolate the sample concentrations.

### 2.11. Metabolomic Analysis

Liquid samples were prepared for ^1^H‐NMR analysis using a JANUS G3 automated liquid handler (Revvity, USA). Samples (449 µL) were mixed with 51 µL of sodium 3‐(trimethylsilyl)propionate‐d_4_ (TSP) dissolved in deuterium oxide (D_2_O), resulting in a final TSP concentration of 1.055 mM. The mixture was transferred to 5 mm NMR tubes and sealed with QR‐coded caps. Samples were stored at 4°C until NMR acquisition.

All ^1^H‐NMR spectra were acquired at 310 K using a Bruker Avance III DRX 600 spectrometer (Bruker GmbH, Rheinstetten, Germany) equipped with a triple resonance probe (1H/ 13C/31P) and operating at 600. 13 MHz for proton detection. A single‐pulse experiment with water presaturation (1 s during the relaxation delay) was used to suppress the solvent signal in all samples. Each spectrum was acquired using 64 scans that collected over 65,000 data points, with a spectral width of 14 ppm [22].

The raw data were processed using MestReNova 8.1 (Mestrelab Research, Santiago de Compostela, Spain), including manual phase correction, baseline adjustment, and chemical shifting with reference to the TSP signal. Metabolite spin systems and resonances were identified and quantified based on previously reported data and the commercial Chenomx NMR Suite Profiler resonance database (Chenomx NMR Suite 8.1, Chenomx Inc., Edmonton, AB, Canada). The processed spectra were exported to MATLAB R2013a (MathWorks, Natick, MA, USA) for further statistical analysis using in‐house scripts.

### 2.12. Proteomic Study

Fresh chondrogenic differentiation medium (as control) and early secretome were analyzed using the Qubit assay (Thermo Fisher Scientific, Waltham, MA, USA). The protein concentration was adjusted to 5 µg for direct in‐solution digestion. Proteins were first reduced with 2 mM dithiothreitol (DTT) in 50 mM ammonium bicarbonate (ABC) for 20 min at 60°C and subsequently alkylated with 5.5 mM iodoacetamide (IAA) in 50 mM ABC for 30 min at RT in the dark. Samples were then digested overnight at 37°C with 250 ng of sequencing‐grade trypsin (Promega Biotech Iberica S.L., Madrid, Spain). After digestion, the reactions were quenched by acidification, and the peptides were dried using a SpeedVac concentrator.

For depletion and enrichment of low‐abundance proteins, the remaining sample volume was processed using the Enrich IST kit (PreOmics GmbH, Martinsried, Germany) according to the manufacturer’s protocol. Briefly, 250 µL of the medium was dried, resuspended in 20 µL of 50 mM ABC, and subjected to the kit protocol. Peptides digested with both approaches were resuspended in 25 µL of 0.1% formic acid in water, and 200 ng were loaded onto Evotip Pure tips (Evosep A/S, Odense, Denmark) following the manufacturer’s instructions.

Peptide separation and analysis were performed using a timsTOF fleX mass spectrometer (Bruker GmbH, Rheinstetten, Germany) operated in diaPASEF mode, coupled to an Evosep One system with a 30‐samples‐per‐day (SPD) gradient (45 min). Peptides were separated on a 15 cm × 150 µm C18 analytical column (1.5 µm particle size) and ionized using a CaptiveSpray source at 1700 V and 200°C. Mass spectrometry (MS) data were acquired with custom diaPASEF settings: 1/K0 ion mobility range of 0.6–1.6 V·s/cm^2^, 100 ms ramp time, and 100–1700 m/z scan range in the positive mode.

Data were processed with DIA‐NN v1.8 using an in silico predicted spectral library generated from the UniProt/SwissProt human proteome database. Protein quantification was based on unique values from gene groups filtered with a false discovery rate (FDR) of 1%. For biological interpretation, STRING database analysis was performed to explore functional enrichment and protein–protein interaction networks of differentially abundant proteins [23].

To further study proteins potentially involved in cartilage regeneration in our samples, a subset of proteins with statistically significant differences in abundance between the early secretome and the fresh medium was selected and analyzed using the STRING database to explore their association with biological processes relevant to chondrogenesis. Functional enrichment was performed using Gene Ontology (GO) terms, and proteins were selected based on their involvement in the following biological processes: chondrocyte differentiation (GO:0002062), cartilage development (GO:0051216), and regulation of cartilage development (GO:0061035).

### 2.13. Fluorescence Microscopy Analysis of COL1A1, COL2A1, ACAN, and F‐Actin

The expressions of COL1A1, COL2A1, and ACAN were evaluated by IF in cultured cells using specific antibodies, both in 2D cultures and in cells embedded in 3D beads. Cells were fixed with 4% paraformaldehyde in PBS (pH 7.4) for 30 min. They were subsequently washed with PBS and permeabilized with 0.1% Triton X‐100 in PBS for 5 min. After three washes with PBS, cells were incubated for 20 min with a blocking solution (1% bovine serum albumin, BSA, in PBS).

Cells were incubated overnight at 4°C with the appropriate primary antibody diluted in antibody diluent solution as follows: 1:100 for COL1A1 (rabbit polyclonal IgG, HPA011795, Atlas Antibodies, Stockholm, Sweden), 1:500 for both COL2A1 (mouse monoclonal IgG, ZRB1201, Merck KGaA, Darmstadt, Germany), and ACAN (mouse monoclonal IgG1, MA3‐16888, Thermo Fisher Scientific, Waltham, MA, USA). After three washes, cells were incubated for 2 h at RT in the dark with the corresponding FITC‐bounded secondary antibody: anti‐rabbit for COL1A1, anti‐mouse for both COL2A1, and ACAN (all from Sigma‐Aldrich, Merck KGaA, Darmstadt, Germany), all diluted 1:200 in PBS.

F‐actin microfilaments were simultaneously visualized with rhodamine‐conjugated phalloidin (1:400 in PBS; Molecular Probes, Thermo Fisher Scientific, Waltham, MA, USA), while the nuclei were counterstained with DAPI (1:100 in PBS). Both reagents were added together with the secondary antibody and incubated for 2 h at RT in the dark as described above. After incubation, the samples were washed with PBS, mounted, and analyzed using a Leica DM2500 fluorescence microscope (Leica Microsystems, Wetzlar, Germany).

### 2.14. Ultrastructure Analysis by Transmission Electron Microscopy (TEM)

The ultrastructure of the cell‐embedded beads was analyzed by TEM. After 4 weeks of culture, beads were washed with Sorensen’s phosphate buffer (PB) and fixed in 2.5% glutaraldehyde at 4°C overnight. Following fixation, samples were rinsed with PB and postfixed in 1% osmium tetroxide for 2 h at 4°C. Dehydration was performed through a graded acetone series, ending with 100% acetone supplemented with copper sulfate (SO_4_Cu). The specimens were subsequently immersed in propylene oxide, embedded in EPON TM 58005 epoxy resin, and sectioned into ultrathin slices. Sections were double‐stained with 3% uranyl acetate and lead citrate to enhance the contrast. Ultrastructural images were acquired using a TEM (Hitachi HT7800, Japan).

### 2.15. Data Presentation and Analysis

Each replica was obtained from a different batch of cells. All measurements were carried out in triplicate unless otherwise indicated. Quantitative data are presented as means ± SD and were subjected to ANOVA, followed by Tukey’s multiple comparison test (GraphPad Software Inc., San Diego, CA, USA). Significance was accepted at *p* <  0.05.

## 3. Results

### 3.1. First Phase: Optimization of Bead Manufacturing System

#### 3.1.1. Bioreactor Design and Alginate/Agarose Beads Generation

**Figure 3:**
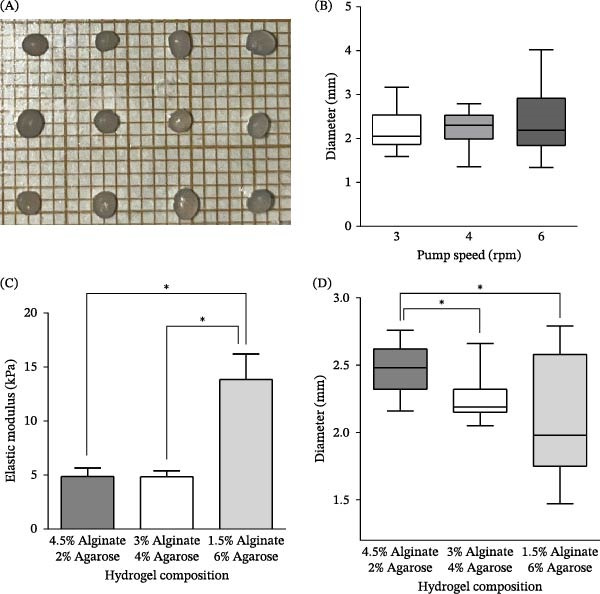
Characteristics of the produced alginate–agarose beads. A bioreactor was designed to optimize bead fabrication. Panel (A) shows representative image of beads generated at a speed of 799 with a polymer concentration of 3% alginate–4% agarose. Panel (B) shows differences in the diameter of the produced beads, according to the speed of the peristaltic pump used. Panel (C) shows variations in the elastic modulus in relation to the hydrogel composition. Panel (D) shows variations in the size of the beads obtained with different alginate‐agarose concentrations. Mean ± SD of *n* = 5 is represented.  ^∗^ *p* ≤  0.05.

First, we designed a bioreactor to produce the alginate–agarose beads as homogeneously as possible, thus minimizing experimental variability. The design of this bioreactor is described above. By modifying the pump speed, we achieved variations in the diameter and morphology of the beads. Three speeds were tested (599, 799, and 999, corresponding to ~3, 4, and 6 rpm, respectively). Speed 599 (3 rpm) was too slow and induced the agarose to gel, clogging the system, so it was discarded. Speed 999 (6 rpm) generated highly heterogeneous beads, while speed 799 (4 rpm) produced the most regular ones (Figure 3, panels A, B). Therefore, this pump speed was selected as the optimal speed for subsequent experiments.

The alginate–agarose concentration was then optimized for bead production. Three different polymer combinations were studied: 4.5% alginate–2% agarose, 3% alginate–4% agarose, and 1.5% alginate–6% agarose, and the elastic modulus was checked. Beads produced with 1.5% alginate and 6% agarose exhibited the highest elastic modulus (Figure 3, panel C) [24]. However, this polymer combination presented considerable challenges in terms of handling and reproducibility. Although the other combinations had lower elastic moduli, the 3% alginate–4% agarose combination produced beads with the smallest average diameter (Figure 3, panel D). Furthermore, this formulation demonstrated improved workability and high reproducibility in the bead generation. Therefore, this combination of 3% alginate and 4% agarose was selected as the most suitable for subsequent experimental procedures.

Once the experimental variables were optimized (pump speed and polymer concentration), our bioreactor was able to produce an average of 130 ± 5 beads per mL of hydrogel, with an average bead diameter of 2.0 ± 0.6 mm. The peristaltic pump used in the manufacturing process had a 1 mm inner diameter tube measuring 55 cm in length. The alginate–agarose solution was extruded at an approximate flow rate of 350 µL/min, allowing rapid and efficient production of ~50 beads/min.

### 3.2. Second Phase: Optimization of Culture System

#### 3.2.1. Bioreactor Design and High‐Density Cells Culture

Next, we designed another bioreactor for the intensive culture of cell‐embedded beads to scale up their production. The design of this bioreactor is detailed in the methodology section (Figure 2, panel B). In this study, we used two peristaltic pumps that allowed us to simultaneously culture four flasks. Considering that we placed ~300 beads in 100 mL of culture medium in each flask, this represents a total of 1200 beads and 400 mL of culture medium. The medium exchange rate was 10% of the total medium, allowing us to collect 40 mL per day. We maintained cell cultures for up to 4 weeks, thus collecting a total of 1120 mL of culture medium from each secretome batch.

**Figure 4:**
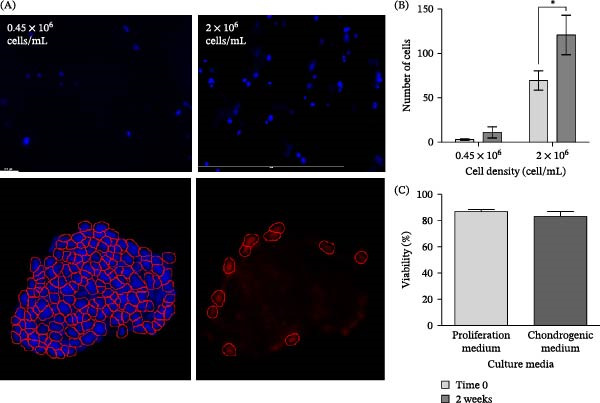
Study of cell density and viability in alginate–agarose beads. hDPSCs were embedded in 3% alginate–4% agarose beads at 0.45 × 10^6^ and 2 × 10^6^ cells/mL and cultured for 2 weeks. Cell number was estimated by DAPI staining, while cell viability was analyzed using the dead/live cell kit. Two time points were considered: time 0 and 2 weeks of culture. Twenty fields from different beads and each experimental condition were acquired with a 10× magnification objective. Panel (A) shows cells freshly seeded (time 0, top) or cultured for 2 weeks in chondrogenic differentiation medium (bottom). Panel (B) shows cell density at time 0 and at 2 weeks of culture. Panel (C) shows viability of cells embedded in beads, cultured with proliferation or chondrogenic differentiation media for 2 weeks. Data are represented as mean ± SD of *n* = 5 independent experiments.  ^∗^ *p* ≤  0.05.

To produce hDPSCs‐embedded beads, the cells were first expanded in monolayer, in a culture with MEM‐proliferation medium, until confluence, then detached with trypsin, and finally suspended in the alginate‐agarose solution at 37°C to make the beads. Two cell densities were evaluated: 0.45 × 10^6^ and 2 × 10^6^ cells/mL. To optimize the cell density in the beads, we cultured them in the bioreactor for 2 weeks in a chondrogenic differentiation medium. The number of cells in the beads was estimated by microscopy, counting the number of DAPI‐positive nuclei in 20 fields from different beads, acquired with a 10× magnification objective. The results obtained are summarized in Figure 4, panels A and B. Initially (time 0), isolated nuclei were observed to be homogeneously distributed throughout the bead (Figure 4, panel A, top). 2 weeks later, the cell distribution had changed, and large, tightly cohesive cell clusters could be observed throughout the beads (Figure 4, panel A, bottom), whereas the cell population within the beads was doubled in both cell densities analyzed (Figure 4, panel B). Then, we studied the viability of the cells cultured in alginate–agarose beads for 2 weeks, both in proliferation and in chondrogenic differentiation media. Cell viability was estimated using the live/dead cell assay kit, as described in the Methods section. The results obtained are summarized in Figure 4, panel C and are represented as the percentage of live cells in 20 fields from different beads, obtained with a 10× magnification objective. As can be observed, no significant cell death was detected in any of the media analyzed.

Therefore, we decided to use the initial concentration of 2 × 10^6^ cells for subsequent experimental procedures since, after 2 weeks of culture, they showed a high proliferation rate with a low cell mortality rate.

#### 3.2.2. Chondrogenic Induction of hDPSCs Embedded in Hydrogel Beads

**Figure 5:**
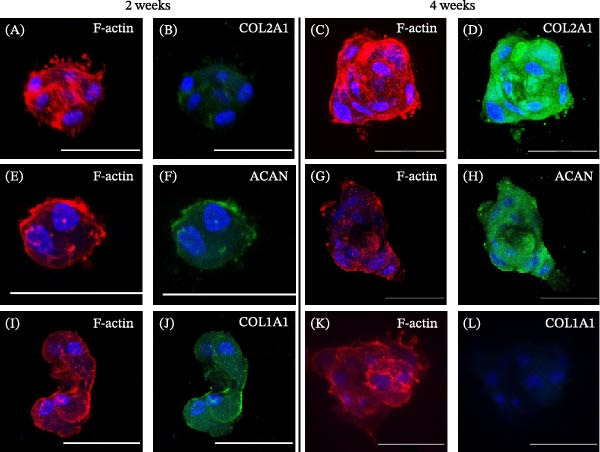
Chondrogenic differentiation of cells embedded in hydrogel beads. hDPSCs were embedded in 3% alginate–4% agarose beads and cultured for up to 4 weeks. The content and distribution of F‐actin (panels A, C, E, G, I, and K) were studied by F‐actin fluorescence staining with phalloidin‐rhodamine. The content and distribution of COL2A1 (B and D), ACAN (F and H), and COL1A1 (J and L) were studied by IF. The images shown are representative of *n* = 5 different experiments. The scale bar is equal to 50 µm.

Our experimental design included the culture of hDPSCs‐embedded beads in a chondrogenic differentiation medium to stimulate the release of chondrogenic factors. To this end, beads with embedded hDPSCs were cultured for up to 4 weeks, and the expression of COL1A1, COL2A1, and ACAN was evaluated by IF at 2 and 4 weeks of culture. Cytoskeletal organization was also assessed by fluorescent F‐actin staining. Figure 5 shows representative images.

The cells acquired a rounded morphology and were observed to form abundant small clusters at week 2 of culture, while at week 4, the cell clusters were less numerous but larger and composed of an increased number of cells (Figure 5). Using F‐actin microfilament staining, few stress fibers were observed at any time studied, but F‐actin condensations did appear at the intercellular junctions (Figure 5, panels A, C, E, G, I, and K). The expression of COL2A1 and ACAN was evident by week 2, increasing significantly by week 4 (Figure 5, panels D and H). In contrast, COL1A1 expression decreased to undetectable levels by week 4 (Figure 5, panels J and L).

**Figure 6:**
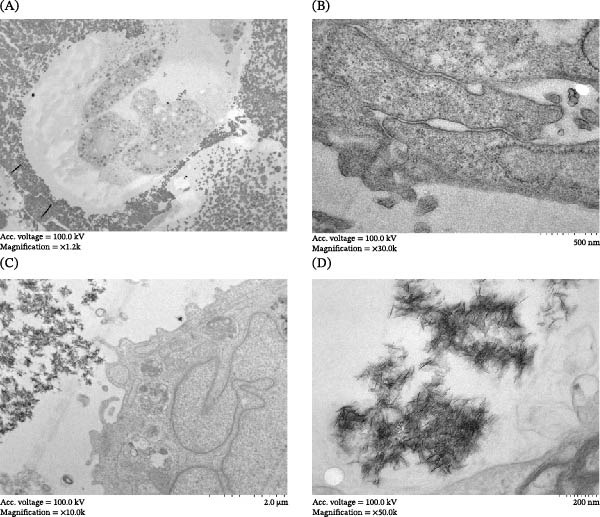
Ultrastructural analysis of cells cultured on the platform, performed using TEM. hDPSCs were embedded in 3% alginate–4% agarose beads and cultured for 4 weeks. Panel (A) shows panoramic view of a group of cells. Panel (B) shows detail of the interdigitations between cells. Panel (C) shows periphery of a cell related to collagen fiber deposits. Panel (D) shows magnification of collagen fiber mesh. The images shown are representative of *n* = 5 different experiments.

**Figure 7:**
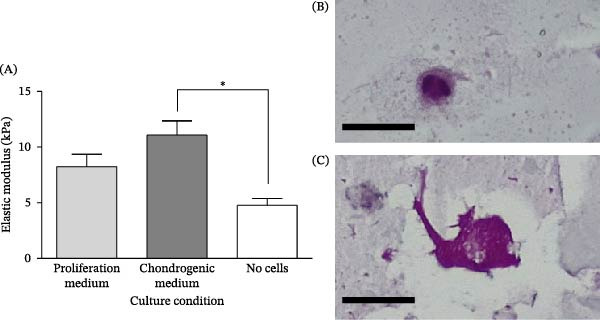
Elastic modulus and PAS staining to detect GAGs secretion in the cells‐embedded hydrogel beads. 3% alginate–4% agarose beads with embedded hDPSCs were cultured in the bioreactor for up to 4 weeks in proliferation or in chondrogenic differentiation media. Panel (A) shows elastic modulus in the studied groups, shown as mean ± SD. PAS staining to analyze GAGs secretion in cells cultured for 2 weeks (panel (B)) or 4 weeks (panels (B and C)). Data are representative of *n* = 5 different experiments.  ^∗^ *p* ≤ 0.05.

These findings were confirmed by TEM (Figure 6). Cells cultured in the bioreactor and stimulated with chondrogenic induction medium organized themselves into compact clusters of interdigitated cells with a secretory phenotype characterized by the presence of secretory vesicles, ERs, and mitochondria. These cells displayed polymorphic nuclei with highly loose chromatin. They also exhibited a cytoskeleton organized in bands at the cell periphery. The cell surface in contact with the biomaterial showed the presence of small microvilli and cellular extensions associated with large deposits of small collagen fibrils organized into meshes. No signs of apoptosis, necrosis, autophagy, or bacterial contamination were observed in the surrounding biomaterial. We next studied the effects of cells on the mechanical properties of the beads and the secretion of glycosaminoglycans (GAGs) into the surrounding hydrogel. We measured the elastic modulus of alginate–agarose beads, with and without cells embedded, cultured for 4 weeks in either proliferation or chondrogenic differentiation media. The results are shown in Figure 7, panel A. As can be observed, the elastic modulus increased significantly in beads with embedded cells and cultured in chondrogenic differentiation medium compared to beads with no cells embedded. PAS staining was performed to analyze GAGs secretion in hDPSCs cultured in chondrogenic differentiation medium for 2 or 4 weeks. As mentioned above, numerous cell clusters were observed in all samples, but they were more numerous in the 2‐week culture experimental group. These cells were PAS‐positive, and GAGs were also detected in the extracellular matrix around the cell clusters (Figure 7, panel B). In the 4‐week culture group, the cell clusters were larger, consisted of a greater number of cells, and some were strongly PAS‐positive (Figure 7, panels B and C).

Therefore, we verified chondral induction in cells embedded in the hydrogel beads, with the presence of chondral markers that increased with culture time and with the number of cells seeded (2 × 10^6^ cells).

#### 3.2.3. Secretome Collection

We cultured beads containing 2 × 10^6^ hDPSCs/mL for 4 weeks in the bioreactor, and 100x concentrated secretome was collected, as detailed in the Methods section. Aliquots of 1.5 mL were frozen and kept at −80°C until use.

### 3.3. Third Phase: Characterization of Secretome

Once cell culture conditions were optimized, our objective was to determine the presence and content of known chondral differentiation markers in the secretome obtained so that it could be used in in vitro models.

#### 3.3.1. Analysis of Known Markers

**Figure 8:**
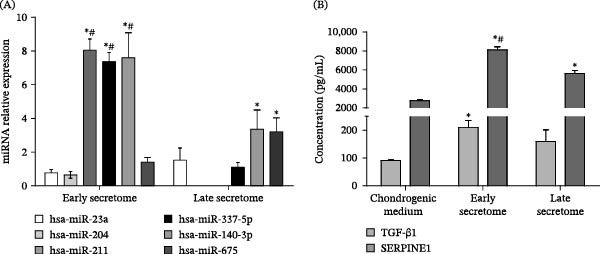
Expression of chondrogenic markers in early and late secretomes. 3% alginate–4% agarose beads containing hDPSCs were cultured in the bioreactor for up to 4 weeks in chondrogenic differentiation medium. The secretome was collected up to 2 weeks (early) and 4 weeks (late) of culture. Panel (A) shows expression of miRNas measured by real‐time RT‐qPCR in early and late secretomes. Panel (B) shows concentration of TGF‐β1 and SERPINE1 proteins quantified by ELISA in early and late secretomes, and in the control culture medium. Data are represented as mean ± SD of *n* = 5 different experiments.  ^∗^ *p* ≤ 0.05 compared to the control group. # *p* ≤ 0.05 compared to late secretome.

Total RNA was extracted from the secretome, and relative gene expression of chondral differentiation markers miR‐23a, miR‐204, miR‐211, and miR‐337‐5p was analyzed by real‐time RT‐qPCR. The results obtained are summarized in Figure 8, panel A. Both experimental groups (early and late secretomes) were compared, and as it is shown, all miRNAs were detected in the early secretome, whereas miR‐23a, miR‐337‐5p, miR‐140, and miR‐675 were present in the late one. Furthermore, the expression of all analyzed miRNAs, except miR‐23a, was significantly higher in the early secretome than in the late one.

TGF‐β1 and SERPINE1 concentrations were analyzed by ELISA, using fresh chondrogenic differentiation medium as control. As summarized in Figure 8, panel B, both proteins were present in all media analyzed, but most importantly, their concentration increased significantly in both early and late secretomes compared to the control medium. TGF‐β1 levels were similar in both secretomes, whereas the SERPINE1 concentration was significantly higher in the early secretome than in the late one.

These data corroborate the presence of chondral differentiation markers, both at the protein and miRNA levels, in the secretomes released by the hDPSCs embedded in hydrogels at weeks 2 and 4 of culture.

#### 3.3.2. Metabolomic and Proteomic Studies

**Figure 9:**
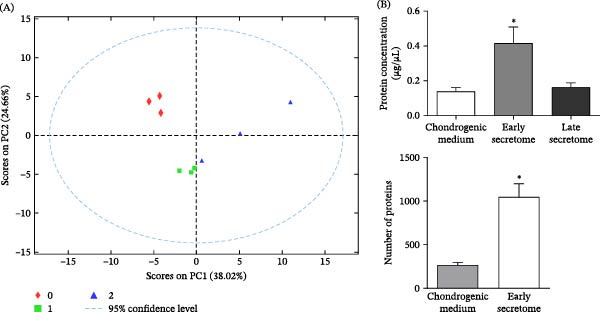
Metabolomic and proteomic characterization of the secretome. hDPSCs‐embedded in 3% alginate–4% agarose beads were cultured in the bioreactor for up to 4 weeks in chondrogenic differentiation medium. Secretome was collected up to week 2 (early) and 4 weeks (late). Fresh chondrogenic differentiation medium was used as control group. Panel (A) shows results of the metabolomic study in the three experimental groups (control group in red, early secretome in green, late secretome in blue, analyzed by PCA. Panel (B) shows protein concentration in the three experimental groups (top), and number of the different proteins in the early secretome and in the control group (bottom). Data are represented as mean ± SD of *n* = 5 different experiments.  ^∗^ *p* ≤  0.05 compared to the control group.

Next, we aimed to further characterize the collected early and late secretomes at the metabolomic and proteomic levels. Metabolomic analysis was carried out by proton ^1^H‐NMR, and an ANOVA test with *p*‐value correction for multivariate models was used to identify differentially expressed metabolites between the early and late secretome. Fresh chondrogenic differentiation medium was used as control. After statistical analysis, a total of 24 metabolites were detected. System variability was studied by principal component analysis (PCA), and the two main variables are represented in Figure 9, panel A. Samples tended to cluster according to variance, dividing into two main groups: the chondrogenic differentiation medium on the one hand and the early and late secretomes on the other. The early secretome samples formed a more cohesive group than the late secretome samples, which showed a greater dispersion. Pathway analysis of the obtained data was performed according to the KEGG Pathway Database, and the metabolites with significantly altered levels are shown in Table 1, which expresses the fold change with respect to the fresh chondrogenic differentiation medium [25]. Twelve metabolites were included in the amino acid biosynthesis pathway. Five of them showed a higher abundance in the early secretome than in the other groups studied, with 2‐oxobutyrate and N‐acetylglutamate being the most notable, reaching ratios of 5.45 and 2.83, respectively. Of the remaining metabolites, 2‐aminoisobutyric acid, imidazole, and glutamine showed a significant decrease. Five other metabolites were altered: two from the glycolysis or gluconeogenesis pathway and three from the methane metabolism pathway, standing out as trimethylamine and formate, with increases in both experimental groups.

**Table 1:** Metabolomic analysis of the collected secretomes.

Metabolite	Fold change		*p*‐Value		Pathway
	Early secretome	Late secretome	Early secretome	Late secretome	
Choline	1.03	0.86	0.1950	0.0640	Biosynthesis of amino acids
Glycine	0.62	0.78	0.0008	0.0007	
Threonine	1.12	1.27	0.0540	0.0210	
2‐Oxobutyrate	5.45	4.22	0.0140	0.0380	
2‐Aminoisobutyric acid	0.65	0.59	0.0040	0.0010	
Imidazole	0.36	0.30	0.0260	0.0010	
Glutamine	0.44	0.29	0.0040	0.0020	
Pyroglutamate	1.57	1.75	0.0001	0.0001	
Histidine	0.90	0.86	0.0100	0.0050	
N‐Acetylglutamate	2.83	1.46	0.0030	0.4510	
Tryptophan	0.88	1.00	0.0250	0.9540	
Pyruvate	0.86	0.94	0.0010	0.3170	
Lactate	0.68	1.60	0.1780	0.0050	Glycolysis or gluconeogenesis
Pyruvate	0.86	0.94	0.0010	0.3170	
Trimethylamine	2.30	5.85	0.2600	0.0110	Methane metabolism
Formate	2.53	6.49	0.0001	0.0530	
Methanol	0.86	0.43	0.0010	0.0340	
3‐Methylxanthine	0.63	0.61	0.0030	0.0130	Caffeine metabolism
Dimethylmalonic acid	0.45	0.76	0.0001	0.2320	Fatty acid biosynthesis
N‐Acetylmannosamine	0.45	0.60	0.0090	0.0430	Biosynthesis of nucleotide sugars
Orotidine	0.15	0.26	0.0001	0.0001	Pyrimidine metabolism
Pseudoephedrine	1.29	0.60	0.2340	0.0040	Biosynthesis of various alkaloids
Raffinose	0.18	0.17	0.0001	0.0001	Galactose metabolism

Before proteomic analysis was performed, protein concentrations were measured in the collected secretomes. The results are summarized in Figure 9, panel B, top. As shown, protein concentrations were significantly higher in the early secretome group, whereas the control group (fresh chondrogenic differentiation medium) and late secretome group reached similar values.

Based on these metabolomic results and those from miRNAs of known markers, we decided to perform the proteomic study only in the early secretome group (up to 2 weeks of culture). Protein analysis was performed as described in the Methods section.

The number of different proteins identified was 4 times higher in the early secretome group than in the control one (Figure 9, panel B, bottom). Then, a targeted proteomics analysis was performed by querying the STRING (functional protein association networks) database using the following keywords: chondrocyte differentiation, cartilage development, and regulation of cartilage development. We only considered those proteins that were detected in at least three of the four samples analyzed for each condition. A total of 203 proteins were selected, of which 51 were detected in the early secretome, whereas only 19 were detected in the control group, all of them markedly decreased compared to the early secretome group. The results obtained are summarized in Table 2.

**Table 2:** Proteomic analysis of the early secretome compared to the chondrogenic differentiation medium.

Protein	Concentration (µg/µL)		Fold change	*p*‐Value	Pathway
	Early secretome	Chondrogenic medium			
ALDH1L2	—	54,800	—	—	Cartilage development
CD109	11,071	—	—	—	
CILP	16,093	—	—	—	
HSPA5	272,887	58,628	4.65	0.0068	
MMP1	41,378	—	—	—	
MMP14	26,818	—	—	—	
MMP2	540,151	59,925	9.01	0.0001	
MMP7	153,300	—	—	—	
NT5E	44,681	—	—	—	
OGN	12,882	—	—	—	
PRG4	19,604	—	—	—	
SERPINA10	13,878	—	—	—	
SERPINA7	37,107	—	—	—	
SERPINC1	300,974	13,518	22.27	0.0001	
SERPINE1	428,771	15,731	20.73	0.0139	
SERPINF1	540,373	11,630	46.46	0.0001	
SERPINF2	417,481	—	—	—	
SERPING1	18,779	—	—	—	
SERPINH1	15,595	—	—	—	
SLC2A1	35,401	—	—	—	
STC1	5511	—	—	—	
THBS3	58,238	—	—	—	
TIMP1	112,189	—	—	—	
TIMP3	20,602	—	—	—	
TNC	23,942	—	—	—	
COL10A1	75,610	—	—	—	Cartilage development and chondrocyte differentiation
COL11A1	11,025	—	—	—	
COL12A1	82,366	33,146	2.48	0.0011	
COL18A1	32,889	—	—	—	
COL1A1	378,412	156,495	2.42	0.0088	
COL1A2	538,348	265,115	2.03	0.0067	
COL2A1	866,681	—	—	—	
COL3A1	221,387	36,811	6.01	0.0001	
COL4A1	8271	27,336	0.30	0.0678	
COL5A1	84,707	21,281	3.98	0.0001	
COL5A2	49,073	21,260	2.31	0.0333	
COL6A1	465,159	116,806	3.98	0.0084	
COL6A2	156,475	27,019	5.79	0.0049	
COL6A3	529,608	51,184	10.35	0.0005	
COL8A1	110,993	—	—	—	
COL9A1	17,768	—	—	—	
COMP	33,316	12,632	2.64	0.003	
ECM1	48,390	—	—	—	
TGFBI	1,583,778	65,577	24.15	0.0003	
CTSK	24,290	—	—	—	Regulation of cartilage development
EFEMP1	13,472	—	—	—	
GLG1	11,983	—	—	—	
GREM1	7569	—	—	—	
LOXL2	9969	—	—	—	
MDK	3468	—	—	—	
TGFB1	548,800	38,189	14.37	0.0029	

Thus, several proteins related to cartilage development, chondrocyte differentiation, and regulation of cartilage development were induced in our model since a high increase in their concentration was observed in early secretome samples with respect to fresh chondrogenic differentiation medium, which presented only a few of these proteins, and at a very low concentration.

### 3.4. Fourth Phase: Validation of Secretome

#### 3.4.1. Protein Expression Analysis of COL1A1, COL2A1, and ACAN

**Figure 10:**
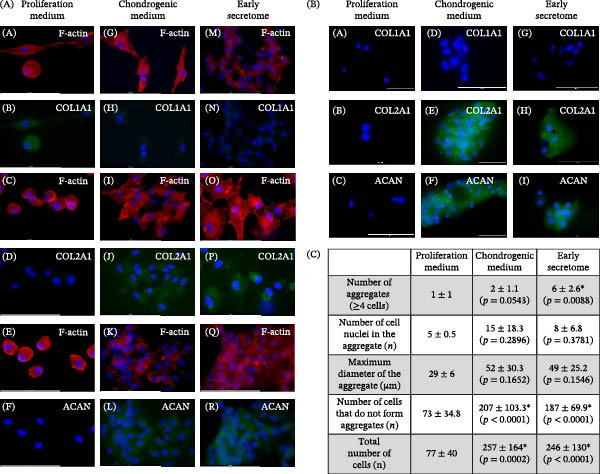
Evaluation of the chondrogenic effect of the secretome in vitro, after 4 weeks of culture. The early secretome was used to induce chondral differentiation in primary cultures of human chondrocytes, cultured in 2D (panel (A)) or 3D (panel (B and C)). Cells cultured with proliferation or chondrogenic differentiation media were used as negative and positive controls for differentiation, respectively. Panels (A and B) shows expression of types I and II collagen, as well as aggrecan, evaluated by IF (green). Cell morphology was studied by fluorescent staining of F‐actin with phalloidin‐rhodamine (red). Nuclei were stained with DAPI (blue). Panel (C) shows morphometric analysis of cell distribution in 3D cultures. Data shows representative results for *n* = 5 different experiments.  ^∗^ *p* ≤ 0.05 compared to the proliferation medium.

Finally, we evaluated the chondrogenic properties of the collected secretome. Human primary chondrocytes were cultured in 2D and exposed to either the early secretome, the chondrogenic differentiation medium (as a positive control), or the proliferation medium (as a negative control) for 4 weeks. The expression of COL1A1, COL2A1, as well as ACAN, was studied by IF, while cell morphology and organization were analyzed by fluorescent staining with F‐actin. Results are summarized in Figure 10, panel A. Chondrocytes cultured in proliferation medium expressed significant levels of type I collagen, while they did not express either type II collagen or aggrecan. Cells grew dispersed on the culture plate without showing interactions with other cells. When chondrocytes were cultured with a chondrogenic differentiation medium or with a secretome, an increase in the number of cells was observed, forming cohesive cell clusters. No expression of type I collagen was detected in these groups, while the expression of type II collagen and aggrecan was significantly increased. Furthermore, these changes were higher in cells exposed to the secretome than in those cultured with chondrogenic differentiation medium.

Results obtained with the 2D cultures were validated in a 3D culture model consisting of primary cultures of human chondrocytes embedded in an alginate–agarose hydrogel and cultured with the collected early secretome for 4 weeks. Furthermore, the chondrocyte‐embedded hydrogels were cultured with either proliferation or chondrogenic differentiation culture media, which served as negative and positive differentiation controls, respectively. The expressions of COL1A1, COL2A1, and ACAN were studied by IF (Figure 10, panels B and C). As can be observed, the early secretome induced the expression of type II collagen and aggrecan, similarly to the chondrogenic differentiation medium (used as a positive control), and induced cells to organize into large aggregates in both groups. However, cells cultured in proliferation medium (negative control) did not form clusters but were mostly isolated, and no positive immunostaining was observed for any of the markers studied.

Morphometric analysis of 3D cultures was performed, and the results obtained are summarized in Figure 10, panel C. As shown, both the chondrogenic differentiation medium and the early secretome significantly increased the number of cells in the hydrogel. Significantly, the secretome induced the formation of a greater number of cellular aggregates that reached a maximum diameter that was almost twice the diameter of the cell aggregate in the control group, cultured with proliferation medium.

## 4. Discussion

Articular cartilage regeneration is an extremely complex process determined in part by the balance between proinflammatory and regenerative biochemical molecules that constitute the tissue microenvironment. MSCs actively participate in this balance by secreting various factors that modulate this microenvironment and generate a friendly environment, promoting the replacement of damaged tissue with new tissue that has biomechanical properties compatible with its cushioning function. Otherwise, fibrous tissue is generated, but it is unable to perform these functions and thus can lead to joint failure [26].

Based on these facts, many strategies are being used to stimulate articular cartilage regeneration using MSCs combined or not with scaffolds, which attempt to provide temporary biomechanical support, which is essential for the successful regenerative process. Although the use of MSCs is widely accepted as a key element to induce the regenerative process, their application in clinical practice is extremely complex due to factors related to limited expansion, poor stability, immunocompatibility, differentiation, heterogeneity, or rapid migration to other organs such as the lung, which can cause thrombosis [27–29]. Therefore, various research groups are working on the hypothesis of using the products secreted by these cells, the secretome, as a pharmacological tool for the treatment of pathologies affecting articular cartilage [30–32].

For the MSCs secretome to be used as a pharmacological tool, it is necessary to standardize some relevant aspects of its manufacture, such as the source of MSCs, the culture conditions, the mechanism of induction of MSCs to secrete the desired factors, the development of intensive culture systems that allow obtaining large quantities of secretome, and, finally, the characterization of its components in order to establish biomarkers that allow us standardizing its use.

Regarding the source of MSCs, bone marrow‐derived MSCs (BM‐MSCs) should be the cell of choice as they are the ones that migrate in vivo to injured cartilage and organize its regeneration. This is, at least, the basis of various therapeutic strategies used in clinical practice, such as microfracture [32]. However, their use presents some limitations related to their inherent heterogeneity and limited expansion capacity compared to that of MSCs from other origins [33]. This low proliferative capacity makes it difficult to use in intensive culture systems. In fact, as a first approach, we attempted to use BM‐MSCs but were unable to expand the cultures to the cell counts required for our approach to produce large amounts of the secretome (data not shown). Another alternative is adipose‐derived stem cells (ADSCs), which offer significant advantages in terms of their origin that would facilitate the use of autologous cells. However, their proliferative capacity is also limited, and they present greater variability, also related to the tissue from which they are obtained [33, 34].

Thus, we chose to use hDPSCs not only because of their chondrogenic differentiation potential, demonstrated both in vitro and in vivo models of osteochondral damage but also because of their greater proliferative capacity [8, 9, 14, 34, 35]. This is essential for establishing a high‐density culture at the scale we have worked with. This fact was not an obstacle with hDPSCs, which have demonstrated enormous proliferation capacity without losing their self‐renewal and differentiation properties [36].

Several authors have established the importance of stimulating MSCs to secrete the factors necessary to induce regeneration [37]. In our case, we have explored three important variables for the induction of the chondral phenotype: the use of an appropriate induction medium, culture under hypoxic conditions, and the use of a hydrogel for 3D culture that, on the one hand, acts as an inducer of differentiation into chondrocyte‐like cells and, on the other, allows for high‐density cell culture. The secretome composition is dynamic and changes during chondrogenesis. For example, in equine models, the level of angiogenic factors has been observed to decrease when MSCs differentiate towards chondrogenic or osteogenic lineages, which contributes to maintaining cartilage avascularity after tissue regeneration [38, 39]. To recreate the tissue microenvironment, we cultured cells in a widely used chondrogenic differentiation medium, which contains known biochemical factors involved in the regeneration process, such as ITS, TFGβ and dexamethasone [8, 9, 14]. Also, we used an alginate/agarose hydrogel that not only contributes to generating a hypoxic environment necessary for chondral differentiation but also induces differentiation to this phenotype by inhibiting the binding contacts between MSCs and the culture surface [40, 41].

The main challenge of this work was to develop a bioreactor capable of providing standardized culture conditions to produce large amounts of the secretome with minimal variability. hDPSCs were cultured in hydrogel beads to increase the surface area and improve secretion. The bead size was optimized to maintain cell density while allowing proper cell behavior, and conditions were set to rapidly generate many beads. A hydrogel of 3% alginate and 4% agarose with a pump speed of 799 (4 rpm) produced homogeneous ~2 mm beads at ~130 beads/mL and 350 µL/min. Under these conditions, MSCs were cultured at 2 × 10^6^ cells/mL. Since native cartilage has ~10^7^ chondrocytes/mL [42], beads were cultured in a proliferation medium for 1 week before differentiation. This increased cell density fivefold, reaching values similar to native cartilage without affecting viability. The bead elastic modulus was 5 kPa, lower than native cartilage (0.24–1 MPa) [43, 44]; although this did not impair differentiation, stiffness remains a key factor to improve in the model. We also designed and manufactured a bioreactor that would allow us to scale up the secretome production. We generated a simple model based on a stirring system and a daily 10% renewal of the culture medium. Given the importance of hypoxia in the chondral cell differentiation process, we decided to exclude any oxygenation system, which greatly simplified the design. In this regard, the complex biology of chondrocytes proved to be an advantage. Our prototype allowed us to work with 100 mL of culture medium and 300 beads per flask, but we could easily scale it up by either increasing the volume of the flasks or the number of flasks in the bioreactor. hDPSCs cultured in the bioreactor differentiated into chondrocyte‐like cells, forming clusters of rounded cells, with F‐actin microfilaments arranged at their periphery, which express ACAN and type II collagen [8, 9]. These cells produced a significant increase in Young’s modulus according to their extracellular matrix secretory nature [45]. To concentrate the secretome, we decided to use Amicon Ultra centrifugal filters 30 kDa. These filters were chosen due to their restrictive capacity to recover most of the proteins involved in regeneration while also allowing the removal of small molecules, such as salts and metabolites. Regarding key components of the secretome, Lee et al. [46] identified proteins consistently secreted by MSCs, including SERPINE1 (UniProt P05121, ~45 kDa), MMP‐2 (UniProt P08253, ~72 kDa), MMP‐9 (UniProt P14780, ~92 kDa), and extracellular collagens such as COL1A1 (UniProt P02452, ~138 kDa). All of them exhibit molecular weights above 30 kDa. TGF‐β1 (UniProt P01137) is predominantly secreted in its latent complex form (LAP/LTBP‐associated), resulting in an apparent molecular weight exceeding 30 kDa and thus would also be retained in ultrafiltration [47]. Moreover, the concentration of TGF‐ β1 in the two filtered secretome fractions was measured by ELISA, and similar concentrations were found in both of them (data not shown). Similarly, transcription factors relevant to cartilage development, such as SOX9 (UniProt P48436, ~56 kDa) and RUNX2 (UniProt Q13950, ~57 kDa), also exceed this threshold. In addition, extracellular vesicles (EVs), ranging from 30 to 150 nm (exosomes) to ~1 μm (microvesicles), are far larger than the molecular weight cut‐off (MWCO) limit and are therefore retained in the filter; these vesicles contain additional molecules of interest, making their retention desirable for downstream functional analyses [48]. Overall, while the application of a 30 kDa filter does enrich the MSCs secretome with biologically relevant proteins and vesicularcomponents, one limitation is the concomitant loss of low‐molecular weight cytokines and chemokines (e. g., IL8, ~8–11 kDa), which represents an inherent disadvantage when aiming a more concentrated and functionally enriched preparation. Furthermore, larger pores imply lower resistance, resulting in quicker filtration and higher yields of the solvent and smaller molecules. Thus, 30 kDa filters have the largest pores and, additionally, retain the desired factors.

Once secretome production was optimized, we set out to characterize its composition. To do so, we initially analyzed the expression of known chondral. miR‐140 and miR‐675 are well‐known miRNA involved in chodrogenesis and in the induction of the expression of both COL2A1 and ACAN [49–51]. miR‐23a suppresses relevant inflammatory cytokines implicated in OA (IL17 and IL6), as well as chemokines (MCP1) and MMP‐3, by inhibiting IKKα [49]. miR‐204 and miR‐211 are well‐known regulators of joint homeostasis. Suppression of both molecules has been reported in mice to induce matrix‐degrading proteases in articular chondrocytes and synoviocytes, which stimulates articular cartilage destruction [52]. The expression of miR‐337 decreases significantly during endochondral ossification and is directly associated with chondrogenesis, regulating TGFBR2 and modulating the expression of cartilage‐related genes, such as *ACAN* [53]. In parallel, we analyzed the expression of two key proteins in the chondral regeneration process, TGF‐β1 and SERPINE1 [54, 55]. TGF‐β1 promotes MSCs differentiation into chondrocytes and upregulates chondrogenic genes such as SRY‐box transcription factor 9 (*SOX9*) and *ACAN*. SERPINE1 regulates extracellular matrix remodeling and preserves cartilage avascularity by inhibiting serine proteases. Although both proteins were detected in the chondrogenic differentiation medium, their concentration was significantly higher in the early and late secretome samples, reaching their highest expression in the early samples.

Metabolomic analysis of the secretome revealed the consumption of seven amino acids involved in their biosynthesis pathway. Among these, we highlight glutamine, which regulates chondrocyte biology through different pathways, including epigenetic control (converted into acetyl‐CoA via GLUD1, that promotes histone acetylation in genes such as collagen type II (*COL2*) and *ACAN*, anabolism (through transaminases such as GOT2 that produces aspartate, necessary for the proliferation and synthesis of extracellular matrix components), and the reduction of oxidative stress, given its participation in glutathione synthesis [56, 57]. We also observed the uptake of glycine, histidine, and tryptophan, in agreement with previous data reported in chondrocytes under mechanical stimulation, as well as an increase in 3‐methylxanthine, involved in chondral matrix production through the degradation of *Lats1* mRNA [58, 59]. This would also explain the uptake of pyruvate, indicating the necessary entry of chondrocytes into the Krebs cycle [60]. All these findings together indicate high biosynthetic activity of chondrocytes in the bioreactor, in terms of cell proliferation and secretion of extracellular matrix elements, including type II collagen and GAGs, which is consistent with the experimental data presented. In addition, consistent with the glutamine data, a significant increase in 2‐oxobutyrate and pyroglutamate was observed, both related to the synthesis of glutathione, the most relevant antioxidant metabolite [61–63].

In agreement with these observations, we detected lactate and pyruvate uptake, likely as a metabolic adaptation of cells to the high energetic and biosynthetic demands derived from their proliferative and secretory states in culture [64, 65]. This cannot occur naturally without a minimum supply of oxygen in the culture medium, suggesting that hypoxic conditions in culture are not as restrictive as those of native cartilage. Therefore, one aspect worth reviewing in the culture system we have developed could be the increased degree of hypoxia in the cultures.

The increase in trimethylamine is an interesting finding that further supports the proliferative state of cells in the bioreactor. This metabolite is associated with increased metabolic activity due to its role in the synthesis of phospholipids, such as phosphatidylcholine, an essential component of the cell membrane [66]. This finding could be related to the consumption of dimethylmalonic acid or N‐acetylmannosamine, related to the secretion of GAGs and other components of the cartilage extracellular matrix [67].

Therefore, the metabolite profile obtained indicates that, in line with the experimental data, the cells in the bioreactor are in a proliferative and secretory state, which supports the use of this methodology to evaluate the state of the cells in culture and, consequently, predict the effect of the secretome as a pro‐regenerative agent of articular cartilage.

Therefore, the data obtained from miRNA profiling, the analysis of known markers, and the metabolomic profile allowed us to select the 2‐week culture time as the most suitable for using the secretome as an agent to induce chondrogenic regeneration. Thus, MSCs secrete pro‐regenerative mediators before completing their differentiation, confirming the acute effect of these cells observed in other experimental models [11, 68]. This is relevant because it shortens culture time, minimizing the risk of contamination, and allows for less frequent generation of secretome batches, thus increasing bioreactor efficiency.

Regarding secretome characterization, we aimed to evaluate in the early secretome the presence of protein markers related to cartilage differentiation. Therefore, we used HRMS, as it is not only a much more powerful technique than others used for protein analysis but also more reliable and offers higher resolution than antibody‐based techniques [69]. We compared the protein profile of the secretome with that of the cell culture medium, in which we detected the presence of important mediators of chondrogenic differentiation, such as TGF‐*β*1, some serpins, and different collagens, among others. However, the concentration of all of them was significantly higher in the collected secretome. A surprising finding was the presence of CD109 in the analyzed secretome. This protein initially participates in inhibiting the effects of TGF‐*β* [70]. Nevertheless, we also observed the presence of CILP, a marker of mature chondral matrix expressed in the intermediate area of articular cartilage. The expression of both proteins could indicate that, on the one hand, MSCs secrete components of the extracellular matrix, as it happens, and, on the other hand, precise control of TGF‐β is exerted to inhibit TGF‐β‐mediated chondrocyte hypertrophy [71]. These findings are consistent with those observed for MMPs and TIMPs. We have detected MMP1, MMP2, MMP7, and MMP14 in the early secretome. These are key proteins for matrix remodeling, and given the elevated TIMPs expression, we might suggest that the cultured cells are in an early stage of differentiation, as evidenced by studies with hDPSCs cultured in similar systems, as well as by the experimental data presented [72]. In addition, we detected the presence of different serpins in the secretome, such as SERPINE1, which limits the MMP activity by controlling cartilage matrix degradation, SERPINEH1, a key protein for type II collagen folding and secretion, SERPINEF1, which inhibits angiogenesis, and SERPINEF2, which prevents excessive extracellular matrix degradation [73]. With respect to the profile of collagen proteins studied, we have detected, on the one hand, the presence of collagen characteristic of the chondral matrix such as COL2A1, COL9A1, and COL11A1. However, we have also detected the presence of collagens related to hypertrophy and to the immature cartilaginous matrix as well as other collagens such as COL1A1/COL1A2, COL3A1, COL5A1/2, COL12A1, and other regulatory collagens such as COL6 (6A1–3), COL8A1, COL12A1, and COL18A1. In this regard, it is important to note that hDPSCs naturally secrete collagens, such as type I, and that during differentiation, the expression of this type of collagen shifts to type II. Therefore, we are dealing with a differentiation model at a very early stage, and we are probably observing a phenotypic shift from a more fibroblastic cell population to a more chondrogenic one [9]. This is supported by various factors, such as the high levels of COM found, which enhances the chondral matrix components during chondrogenesis [74], EFEMP1, a fibulin relevant to the organization of the articular cartilage matrix [75], GREM1, a suppressor of chondrocyte hypertrophy [76], TGFBI, a known inducer of MSCs differentiation to chondrocytes [77], and of course TGFB, a known inducer of chondrocyte differentiation and an important inducer of the secretion of key elements of the chondral matrix.

## 5. Conclusions

The data obtained at the genomic, metabolomic, and proteomic levels demonstrate that, on the one hand, the cells are viable on the platform, proliferate, and acquire a chondral‐like phenotype. On the other hand, these cells are in an active secretory state and release proteins into the secretome that altogether define a microenvironment favorable for chondral regeneration. This is further supported by the evidence from the in vitro differentiation model, where we observed that the secretome has the potency to induce the differentiation of undifferentiated chondrocytes into mature cells that secrete key components of the chondral matrix. The incorporation of hDPSCs, thanks to their high proliferation and differentiation capacity into chondrocytes, has enabled the development of a platform with the potential to produce large volumes of secretome of known composition, which is essential for generating a product that can be translated to clinical use. However, it is necessary to continue working with different animal models to confirm the results obtained in vitro, especially considering the complex biology of the cartilage tissue.

## Funding

This work was supported by grant PID2022‐138572OB‐C43 (MM) funded by MICIU/AEI/and by “ERDF A way of making Europe,” by “ERDF/EU”, by the “European Union” or by the “European Union NextGenerationEU/PRTR,” and by grant CIPROM/2023/62 (MM) founded by the Generalitat Valenciana.

## Conflicts of Interest

The authors declare no conflicts of interest.

## Data Availability

The datasets generated during the current study are available in the Figshare repository, DOI: 10.6084/m9.figshare.30251944. The remaining data are included in the manuscript. However, if necessary, we can provide additional data upon request.
